# Incidence of distal ulna fractures in a Swedish county: 74/100,000 person-years, most of them treated non-operatively

**DOI:** 10.1080/17453674.2019.1686570

**Published:** 2019-11-04

**Authors:** Maria Moloney, Simon Farnebo, Lars Adolfsson

**Affiliations:** a Department of Plastic Surgery, Hand Surgery, and Burns, Linköping University;; b Department of Orthopaedics, Linköping University;; c Department of Clinical and Experimental Medicine, Linköping University, Sweden

## Abstract

Background and purpose — Fractures of the distal ulna can occur in isolation or in conjunction with a distal radius fracture. They may result in incongruence and instability of the distal radioulnar joint. We investigated the incidence of distal ulna fractures, whether any fracture types were more common, and the methods of treatment used.

Patients and methods — Data were collected from patients 18 years or older, treated for a fracture of the distal ulna in Östergötland, Sweden, during 2010–2012. Patients were identified in the patient registry. The fractures were classified according to the AO comprehensive classification of fractures.

Results — The incidence of distal ulna fractures was 74/100,000 person-years. The most common fracture type was that of the ulnar styloid Q1 (79%), followed by the ulnar neck Q2 (11%). Rarest was ulna head fracture, type Q4 (1%). Incidental findings were a mean age of 63 years (SD 18), a concomitant distal radius fracture in 92% of the patients and that 79% were caused by falling from standing height. Internal fixation was performed in 30% of the Q2–Q6 fractures. This indicates that most were considered stable without internal fixation or stable after fixation of a concomitant radius fracture.

Interpretation — Our results show that fractures of the distal ulna are not very common, and some fracture types are even rare. There seem to be no consensus on treatment.

Fractures of the distal ulna may result in incongruence and instability of the distal radioulnar joint (DRUJ), which may result in chronic pain or limited forearm rotation (Kvernmo 2014). Fractures of the distal ulna most often accompany a distal radius fracture and in the majority of cases they affect the ulnar styloid process, while fractures of the ulnar head and/or neck are less common (Ring et al. 2004). Distal radius fractures and concomitant fractures of the distal radius and ulna are commonly caused by a fall from standing height on an outstretched arm with extended wrist. Isolated ulna fractures on the other hand are most often caused by a direct trauma to the ulnar border of the wrist (Richards and Deal 2014). Among patients with a Colles fracture, excluding ulnar styloid fractures, 5.6% have a concomitant fracture of the distal ulna (Biyani et al. 1995). Internal fixation of these fractures is typically difficult (Ring et al. 2004) as the distal fragment in most cases is small, consisting to a large extent of metaphysis and has a 270° articular surface.

There is no clear consensus on how fractures of the distal ulna should be treated, and there are currently very few data on healing rate, results of different treatment options, and functional results. This retrospective study investigates the incidence of distal ulna fractures in adults, whether any fracture types are more common according to the AO classification, both in relation to cause of trauma and demographics. We also investigated how frequently operative treatment was the treatment of choice related to the different fracture types.

## Patients and methods

Data were collected for patients treated for a fracture of the distal ulna, isolated or in combination with a fracture of the distal radius, in the county of Östergötland, Sweden, between 2010 and 2012 (430 000 inhabitants). The inclusion criteria were: (1) patients with a fracture of the distal third of ulna from January 1, 2010 to Dececember 31, 2012, (2) being 18 years or older, and (3) residing in the county of Östergötland, Sweden, at the time of injury.

**Figure 1.**
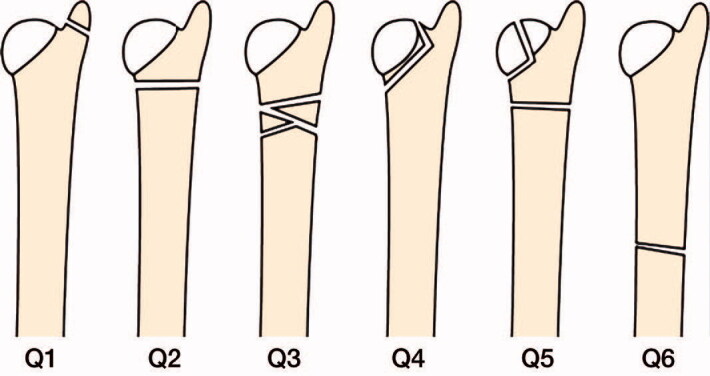
Classification of distal ulna fractures according to the AO comprehensive classification.

For patients who had visited 1 of the 3 emergency units or the 3 orthopedic departments in the area (Linköping University Hospital, Vrinnevi Hospital Norrköping, and Motala Hospital), a diagnosis code according to the ICD-10 system had been recorded in the digital journal system and in the patient registry of Östergötland. In 2015 the patient registry of Östergötland was searched for all ICD-10 codes of a distal forearm fracture (S52.50, S52.51, S52.60, S52.61, S52.20, S52.21, S52.80, S52.81). The radiographs of all patients who had received 1 of these codes were then screened in the digital radiology system to identify everyone who had sustained a fracture of the distal third of the ulna during the defined time period. The cause of trauma and the chosen treatment was also recorded using the referrals and the subsequent radiographs. All images displaying a fracture of the distal third of the ulna but with the exception of styloid fractures were passed on to an independent specialist in radiology for classification. The fractures were classified according to the comprehensive classification of fractures in accordance with the Arbeitsgemeinschaft für Osteosynthesefragen (AO) in which a Q-modifier is used (Müller et al. 1990). Throughout the paper this will be referred to as the AO fracture classification. There are 6 different Q classes of which Q1 refers to styloid fractures (Figure 1).

To examine the reliability of the fracture classification a second specialist in radiology examined blinded the radiographs of 20 randomly chosen patients. The 2 radiologists had been specialists in radiology for 32 and 21 years respectively.

To calculate the incidence of distal ulna fractures we related the number of fractures to the population in the county of Östergötland during 2010–2012 according to the official Swedish statistics, presented by Statistics Sweden (SCB). The mean population aged 18 or older in Östergötland 2010–2012 was 345 971. To calculate incidence of different fracture types in relation to age we subgrouped the examined patient radiographs into 3 categories: 18–34, 35–64, and 65 or older.

### Ethics, funding, and potential conflicts of interest

The study protocol was reviewed and approved by the Regional Ethical Review Board in Linköping (Dnr 2014/200-31). The authors received no financial support, and declare no conflicts of interest.

## Results

We found 766 patients who had sustained a fracture of the distal ulna during the observation period. Of these, 607 had a fracture of the styloid process, and 159 a fracture of the distal third of the diaphysis, metaphysis, or ulnar head (Table 1). The overall incidence of distal ulna fractures in Östergötland was 74/100 000 person-years during 2010–2012.

**Table 1.** Incidence of distal ulna fractures, divided by AO fracture class, in the region of Östergötland, Sweden, during 2010–2012

Fracture class	n (%)	Incidence (/100,000 person-years)
Q1	607 (79)	59
Q2	83 (11)	8
Q3	28 (4)	3
Q4	5 (1)	1
Q5	16 (2)	2
Q6	27 (4)	3
All	766	74

The most common fracture type was a fracture of the ulnar styloid, Q1 (79%), followed by a fracture of the ulnar neck, Q2 (11%). Ulnar head fracture Q4 (1%) was the rarest fracture type. Patients 65 years or older were less likely to have a Q1 fracture when compared with the intermediate and younger groups, as presented in Table 2. Patients aged 65 years or older more commonly than the other age groups had a Q2 fracture (14%) or a Q3 fracture (6%). In patients aged 18–34 years fracture types Q3 and Q4 were not found and only 1 patient had a Q5 fracture (Table 2).

**Table 2.** Amount and proportion (%) of distal ulna fractures, in the region of Östergötland, Sweden, during 2010–2012, divided by age

Fracture class	18–34 years (n = 67)	35–64 years (n = 307)	≥ 65 years (n = 392)
Q1	57 (85)	263 (86)	287 (73)
Q2	4 (6)	25 (8)	54 (14)
Q3	0 (0)	5 (2)	23 (6)
Q4	0 (0)	2 (1)	3 (1)
Q5	1 (1)	5 (2)	10 (3)
Q6	5 (7)	7 (2)	15 (4)
Concomitant radius fracture	58 (87)	281 (92)	372 (95)

The 2 radiologists agreed 70% on classification. The most common disagreement was between Q2 and Q6 where there is no clear landmark defining where the ulnar neck ends and the diaphysis begins. If Q2 and Q6 are considered as the same group the assessors agreed in 90% of the cases. Disagreements were solved by consensus discussion.

The mean age at the time of injury was 63 years (SD 18). 76% of the patients were women. 44% had injured their right wrist. 92% also had a fracture of the radius, almost always the distal radius. Of the fractures in the ulnar head and neck (Q2–Q6) 77% had also sustained a distal radius fracture, compared with 98% of the patients with an ulnar styloid fracture (Q1). Fracture classes Q4 and Q5 were found always to have a concomitant radius fracture whereas only one-third of class Q6 also had a radius fracture. A concomitant distal radius fracture was found in 95% of the patients aged 65 years or older, and in 87% of the patients aged 18–34 years.

The main type of trauma was a fall from standing height on an outstretched arm with an extended wrist, which was the cause in 79% of the patients, followed by fall from a height. The high-energy trauma of road traffic accidents and also blunt trauma including being beaten or kicked always resulted in a simple fracture to the neck or proximal ulna (Q2 and Q6), in no case being comminuted or including the ulnar head (Table 3).

**Table 3.** Cause of injury in distal ulna fractures, in the region of Östergötland, Sweden, during 2010–2012, divided by AO fracture class

Cause of injury	Q1	Q2	Q3	Q4	Q5	Q6	Total (%)
Motor vehicle accident	11	3	0	0	0	5	19 (2.5)
Bike accident	12	2	0	0	0	1	15 (2.0)
Fall from standing height	485	63	27	3	15	11	604 (79)
Fall from height	57	5	0	1	1	2	66 (8.6)
Blunt trauma	16	4	0	0	0	5	25 (3.3)
Forced rotation	3	0	0	0	0	1	4 (0.5)
Unknown	22	5	1	1	0	2	31 (4.0)
No trauma	1	1	0	0	0	0	2 (0.3)

In patients aged 65 years and older the fracture was a result of a fall from standing height in 87%. The second most common cause of injury in this group was fall from a height and all other causes were very rare (0–3%). High-energy trauma was more common in patients aged 18–34 years. Noticeably, a fall from standing height was not found to result in a fracture of class Q2–Q6 in this age group, only fractures of class Q1. The trauma in patients aged between 35 and 64 years, resembled that in the older age group (Table 4).

**Table 4.** Cause of injury in distal ulna fractures, in the region of Östergötland, Sweden, during 2010–2012, divided by age, n (%)

Fracture class	18–34 years (n = 67)	35–64 years (n = 307)	≥ 65 years (n = 392)
Motor vehicle accident	13 (19)	3 (1)	3 (1)
Bike accident	5 (7)	5 (2)	5 (1)
Fall from standing height	27 (40)	237 (77)	340 (87)
Fall from height	7 (10)	31 (10)	28 (7)
Blunt trauma	10 (15)	10 (3)	5 (1)
Forced rotation	1 (1)	3 (1)	0 (0)
Unknown	4 (6)	17 (6)	10 (3)
No trauma	0 (0)	1 (0)	1 (0)

Of all the patients with Q2–Q6 fractures 30% were treated operatively with internal fixation of the ulna fracture. Fracture class Q4 (3/5) and Q3 (12/28) were most commonly surgically treated. Internal fixation was performed in 10 patients with an isolated ulna fracture. Out of these there were 6 Q2, 3 Q6, and 1 Q3 (Table 5).

**Table 5.** Treatment of distal ulna fractures AO class Q2–Q6, in the region of Östergötland, Sweden, during 2010–2012, n (%)

Fracture	Ulna fracture	Concomitant radius fracture	Operated only radius	Operated only ulna	Operated radius + ulna	Total operated ulna
Q2	83	65 (78)	22 (27)	6 (7)	13 (16)	19 (23)
Q3	28	26 (93)	1 (4)	1 (4)	11 (39)	12 (43)
Q4	5	5 (100)	2 (40)	0 (0)	3 (60)	3 (60)
Q5	16	16 (100)	7 (44)	0 (0)	5 (31)	5 (31)
Q6	27	10 (37)	2 (7)	3 (11)	6 (22)	9 (33)
Total	159	122 (77)	34 (21)	10 (6)	38 (24)	48 (30)

All operated ulna fractures that had a concomitant radius fracture also had internal or external fixation of the radius fracture, most commonly a volar plate and screws. In some cases only the radius fracture was internally fixed and the ulna fracture treated non-operatively; for example with fractures of class Q2 and Q5 it was more common only to repair the radius rather than to internally fix both fractures (Table 6).

**Table 6.** Treatment of concomitant fractures of distal radius and ulna AO class Q2–Q6

Fracture	Radius + ulna fracture	Operated ulna only	Operated radius only	Both fractures operated
Q2	65	0	22	13
Q3	26	0	1	11
Q4	5	0	2	3
Q5	16	0	7	5
Q6	10	0	2	6
Total	122	0	34	38

## Discussion

Fractures of the distal ulna are not very common, especially when excluding fractures of the ulnar styloid, and are most often found with a concomitant distal radius fracture. Our findings showed an incidence of 74/100 000 person-years in adults living in Östergötland, Sweden, during 2010–2012. Most common were fractures of the ulnar styloid (79% Q1) followed by fractures of the ulnar neck (11% Q2). Herzberg and Castel (2016) found that, excluding styloid fractures, 9% of patients with a distal radius fracture also have a distal ulna fracture. They defined the distal ulna as the ulnar head and neck without further specification and not the distal third of the ulna as in the present study. 5.9% were a fracture of the ulnar neck, 1.6% a fracture of both the head and neck, and 1.4% a fracture of ulnar head (Herzberg and Castel 2016). Simultaneous fractures of both radius and ulna could negatively affect the outcome by causing problems of incongruence of the DRUJ and increase the risk of nonunion. Styloid process fractures that include metaphysis often heal; however, when the styloid process is fractured separately from the metaphysis it often does not (Biyani et al. 1995).

Knowledge regarding isolated ulna fractures is limited. Williams and Friedrich. (2011) investigated isolated distal ulna fractures retrospectively and could not find any substantial difference between operative and non-operative treatment when it came to bone healing and complications. They did not investigate the functional results, which may be most important to the patient, and the follow-up was only 36 weeks. Cha et al. (2012) studied a cohort of patients above 65 years of age. They found no difference in healing, pain, or function when comparing an operative plate fixation of the ulna fracture or non-operative treatment at the same time as an internal fixation of a radius fracture.

Logan and Lindau (2008) concluded that isolated fractures of the ulnar head with disruption of the articular surface or instability should be operated and internally fixed, preferably after a CT scan. For fractures within 5 cm from the ulnar head that are considered stable, and fractures that become stable after fixation of a concomitant radius fracture, non-operative treatment was recommended. For unstable fractures, on the other hand, operation with open reduction and internal fixation was considered indicated. Logan and Lindau, however, also concluded that the knowledge regarding optimal treatment for intraarticular fractures of the distal ulna is limited. The general recommendation is, however, always to try to restore and keep the ulnar head and DRUJ intact, and only as a last resort resect or replace the ulnar head with a prosthesis.

The typical patient with a distal ulna fracture is a postmenopausal woman who falls from standing height on an extended wrist and suffer a fracture of the distal radius and the ulnar styloid in her left hand. When the ulnar styloid fractures are excluded, the most common fracture is that of the ulnar neck (Q2). Our finding (an incidence of 74/100 000 person-years in adults) can be compared with the incidence of distal radius fractures, which has been reported to be 258/100 000 person-years in a Finnish study from 2010 including patients aged 16 years or older. That study suggests that distal radius fractures are more than 3 times as common as distal ulna fractures (Flinkkila et al. 2011). We extended our search of diagnosis codes to all distal forearm fractures to identify potentially miscoded fractures. We therefore believe that the risk of missing patients who sought medical care for a distal ulna fracture would be small, and it appears unlikely that patients with a fracture have not been to hospital for treatment. Every medical record of a patient seeking medical care undergoes several controls to ascertain that a diagnostic code is reported but the completeness of the registry of diagnose codes in Östergötland has not been validated, which may be considered a potential limitation of our study. Östergötland is a county with more than 2/3 of the population living in cities or densely populated areas and may be considered representative of the majority of the country.

The mechanism of injury is most often a fall from standing height. In the older population this could cause fractures of all classes, whereas in the younger population almost exclusively fractures were of type Q1, indicating that more energy is needed for a fracture of class Q2–Q6 to occur in younger individuals. Not surprisingly we found the distal ulna fractures to be more common in females than males and that the average age was 63. This indicates that, just like distal radius fractures, distal ulna fractures can also at least partially be explained by osteoporosis in postmenopausal women. Isolated ulna fractures were more common in patients 34 years and younger and were more often of fracture class Q2 and Q6 when compared with all age groups.

Only one-third of the ulna fractures were operated, showing that most were considered either stable without internal fixation or stable after a concomitant radius fracture was internally fixed.

Proper classification may be of importance to determine the appropriate treatment and assess the prognosis for the patient. We found the AO classification we used to be incomplete in some respects. This is an obvious limitation to our study, where our radiologists for example found it difficult to separate the classes Q2 and Q6 since there is no distinguishing landmark between the 2. Also, some fractures did not fit well into either class. Often these fractures were multifragmented. Furthermore, the classification does not consider the amount of dislocation, which might be of importance when considering treatment options. Since this study was performed in 2015 the AO classification has been revised and the new version from 2018 addresses the dislocation of fractures of the ulnar neck (Meinberg et al. 2018). Whether or not it covers all distal ulna fractures in a better way is yet to be studied.

A third of the fractures of the distal ulna (excluding the styloid) in our material were operated and internally fixed. We cannot from the present study conclude on appropriateness of the chosen treatment. There is a need for further studies regarding treatment, healing rate, and functional results of fractures of the distal ulna.

All authors have contributed to the study and read and approved the finished manuscript before submission. MM and LA designed the study. MM collected and analyzed the data, and wrote the manuscript. SF and LA contributed with analyzing data and revising the manuscript.
